# Modeling SAOS Yield Stress of Cement Suspensions: Microstructure-Based Computational Approach

**DOI:** 10.3390/ma13122769

**Published:** 2020-06-18

**Authors:** Neven Ukrainczyk, Mareike Thiedeitz, Thomas Kränkel, Eddie Koenders, Christoph Gehlen

**Affiliations:** 1 Institute of Construction and Building Materials, Technical University of Darmstadt, 64287 Darmstadt, Germany; koenders@wib.tu-darmstadt.de; 2 Centre for Building Materials (cbm), Technical University of Munich, 81245 Munich, Germany; mareike.thiedeitz@tum.de (M.T.); thomas.kraenkel@tum.de (T.K.); gehlen@tum.de (C.G.)

**Keywords:** cement paste, rheology, small amplitude oscillatory shear (SAOS), yield stress, mathematical modeling

## Abstract

Two static yield stress models, one known as YODEL and the newly proposed BreakPro, based on inter-particle bond breaking probability, were employed to comparatively simulate the yield stress of cement suspensions, induced by oscillatory rheological tests with small amplitude oscillatory shear (SAOS). This yield stress occurs at a critical strain in the order of 0.01%, and is commonly attributed to the limit of the linear viscoelastic domain, where attractive forces bridge the cement particles and form a flocculated particle network. YODEL is based on van der Waals (vdW) interaction forces to describe the yield stress for flow onset at a critical strain of a few percent, developed for simple non-reactive particulate suspensions. However, due to the high pH and reactivity of cementitious suspensions, their particle interaction forces are much higher than vdW. Therefore, until now, the YODEL adaptations to cementitious suspensions did not explicitly consider the microstructural-based salient feature of the original model, but used it as an implicit fitting parameter to scale the average attractive force. In this paper, the force is inversely estimated using the full power of the two microstructural-based models, presenting a new mathematical tool for investigating the fragility of the rigid percolated structure of cement suspensions. The model parameters were calibrated on measured yield stresses obtained by SAOS measurements in a high-sensitivity rheometer. The estimated forces were found to be 5.57 (BreakPro) and 1.43 (YODEL) times higher than typical van der Waals forces. The YODEL percolation threshold of 21% turned out to be significantly lower than the one found by the BreakPro model (37%). This indicated that BreakPro modeling assumptions are better suited for the description of yield stress at SAOS critical strain than the YODEL model.

## 1. Introduction

Cement paste is a suspension with colloidal particles and colloid-like rheological behavior that possesses viscoelastic properties. At applied shear, cement pastes contain an elastic part and a viscous part. At very low shear, elastic parameters dominate over viscous behavior caused by the particle network structure of the cement suspension, where particles of less than 100 µm are agglomerated due to colloidal and colloid-like attractive forces [1,2,3]. At applied shear, the elastic part decreases, whereas viscous behavior, caused by energy dissipation, increases. The proportion between energy stored in the particle system and energy dissipation determines flow parameters of cementitious suspensions, and thus, concrete. The flow behavior, meanwhile, is crucial for the workability, placement and setting properties of concrete [4,5], thus knowledge about the rheological parameters, i.e., yield stress $\tau_{0}$ and viscosity μ, is crucial for the prediction and adjustment of workability characteristics. Most often, parameters measured during high shear are sufficient parameters for the description of concrete flowability and therefore are implemented in common simulations for concrete flow. Still, these parameters are only sufficient for steady state flow, as cementitious pastes exhibit a non-Newtonian flow behavior, wherefore rheological parameters change especially with the applied shear rate. At rest, the particles flocculate and form a rigid network structure. For a fundamental understanding and subsequent simulation of rheological parameters, interparticle interactions need to be understood, which are dependent on various parameters [6]. After the mixing of cement with water, chemical, as well as physical forces, lead to an immediate flocculation of the cement particles. The percolation time is the time needed for the particles to build up a connected particle network due to the interplay between attractive forces and Brownian motion. Interconnected flocs are called percolation paths. An increasing number of unbroken percolation paths leads to a particle network. With an initial flocculation, i.e., percolation time, the coordination number of interacting cement particles increases until reaching a steady state. After reaching a percolated steady state, having a constant coordination number of cement particles due to their physical attraction, the strength of the network structure still increases due to the ongoing chemical nucleation of C-S-H bridges between cement particles. Rheological parameters subsequently are dependent on the built network structure of the cement particles, the strength of the bridges, which continuously increases with nucleation hydration time and the number of contact points (i.e., coordination number) of cement particles in that structure [6,7].

Still, much research is ongoing on identifying the cement particle interaction forces and subsequently the rheological behavior [8,9]. Roussel et al. [10] hypothesized that the rheology might be dominated by the precipitation of the C-S-H nuclei. This is explained by cement cohesion due to bridging by initial C-S-H nuclei at the nanometer scale. Following this hypothesis, Mostafa and Yahia [11] proposed a semi-empirical kinetic model to mathematically describe the percolation time (physical part) and rigidification rate (chemical part) as a function of microstructural parameters. The model parameters include inter-particle cohesion due to ion correlation forces [12,13] (between C-S-H plates), solid volume fraction, the frequency of collision of Brownian cement particles (predominantly below 1 µm), their average separation distance immediately after disruption, the surface average diameter of cement particles and C-S-H nucleation rate constant. They calibrated their model using oscillatory rheometry with small amplitude oscillatory shear (SAOS) tests within the linear viscoelastic region (LVE) to obtain a continuous measure, i.e., the kinetics of structural buildup at rest. However, apart from kinetics, the modeling analysis included no quantification of the yield stress $\tau_{y}$ of the suspension at its critical strain $\gamma_{l}$ at the end of the linear viscoelastic regime, which can be obtained from SAOS measurements.

This paper provides a link between the quantitative data on yield stress $\tau_{y}$ obtained from SAOS measurements of cement suspensions with adapted mathematical models based on a statistical description of gel microstructure [14,15]. Thus, cement particle cohesive forces directly after the mixing process are quantified (calibrated) to extend such a mathematical tool to probe the rigid (elastic) percolated structure of cement suspensions. It should be noticed that the proposed model approach does not explicitly model the real underlying mechanisms for structural buildup nor the actual origin of the inter-particle forces responsible for the network strength. Still, characteristic inter-particle force values were calibrated on measured yield stresses $\tau_{y}$ at the critical strain $\gamma_{l}$ of the suspensions during SAOS measurements. This contribution will enhance the fundamental knowledge related to the initial structuration of reactive cement suspensions, namely by providing a mathematical relation between the fragility of the percolated network (rigid) structure and essential interaction forces between cement particles.

## 2. Theoretical Framework

### 2.1. Non-Destructive Rheological Investigation

In rheometric oscillatory shear tests, a sinusoidal strain is applied, followed by the subsequent resulting mechanical response as a function of time. Dynamic oscillatory shear tests using high sensitive rheometers can be performed within low strain deformations. Small amplitude oscillatory shear (SAOS) tests are performed within the linear viscoelastic mechanical response, whereas measurements with non-linear mechanical responses at large strains are so-called large amplitude oscillatory shear (LAOS) measurements. SAOS measurements are conducted through the application of very low excitations and thus can be assumed as a non-destructive measurement technique, as long as the applied excitation is too low to break the particle network. When conducting SAOS tests, the sinusoidal amplitude during the measurement is increased starting at a very low strain.

(1) $$\gamma \left(t\right)=\gamma_{0}\cos\left(\omega t\right)$$

where γ(t) is the applied strain with time, which is defined by the cosine of an initial strain $\gamma_{0}$ and the frequency ω with time t. A linear sinusoidal stress response to the applied strain occurs within the non-destroyed linear material regime:

(2) $$\tau \left(t\right)=\tau_{0}\cos\left(\omega t+\delta \right)$$

where τ(t) is the shear stress with time t, $\tau_{0}$ is the amplitude of the sinusoidal shear stress response to the oscillatory strain imposed, ω being the frequency in (rad/s) and δ the phase shift angle. Ideal material behavior would be either fully elastic or fully viscous. If the stress response is proportional to the applied strain, the material can be assumed to be fully elastic with Hookean behavior and the shear modulus G* as a proportionality factor: G* =  τ/γ. As soon as the material starts being destroyed, the storage modulus decreases. The resulting strain is the critical strain $\gamma_{l}$ and the yield stress can be calculated following the definition of the storage modulus for viscoelastic materials (Equation (3)) at the end of the linear viscoelastic region (LVE).

(3) $$\tau_{y}=G^{\prime }\;\gamma_{l}$$

The material is fully Newtonian if the resulting stress is proportional to the strain rate. Generally, materials contain both elastic and viscous parts. The shear modulus then is defined as G* = G′ + *i*G″, where G′ is the storage modulus which corresponds to the elastic material response and G″ is the loss (viscous) modulus which corresponds to the viscous material response. The amplitude sweep of a gel may or may not result in a pronounced G″ maximum where solid behavior is still valid: G′ > G″, which is informative of the gel microstructure (e.g., number of first neighbors) and of the particle characteristics (e.g., compressibility) and surface interaction forces. After the critical strain, where the G″ maximum is exceeded, a further breakage would develop (e.g., attributed to a different origin of the attractive colloidal force) throughout the network up to the crossover point: G′ = G″. The shear stress at the crossover point (G′ = G″) is called the flow stress *τ*_f_. At higher shear, the viscous portion will dominate (G″ > G′) and the material starts to flow. If brittle fracturing occurs, yield stress *τ*_y_ has the same value as flow stress *τ*_f_.

In cement paste, as a highly reactive suspension, elastic and viscous parameters change as soon as water is added to dry cement. The interpretation of SAOS measurements thus gets quite complicated. Roussel et al. [10] proposed the model of two critical strains of cement pastes, where the first critical strain is the small critical strain, measured through the SAOS technique and attributed to the bridging of cement particles by C-S-H bonding. The large critical strain is measured through static yield stress tests using rotational rheometry and is attributed to van der Waals forces and the remaining C-S-H bridges [9,16]. The measured elastic network strength was subsequently correlated to actual particle distances, and thus the water to cement (*w*/*c*) ratio and the effect of adsorbed polymers, which act as superplasticizers. Yuan et al. [17], meanwhile, found that the applied rotational excitation needs to be correlated to the effective agglomerates (to account for the bridging effects of C-S-H nano-particles) and not only to the displacement of cement particles. This simplification originates from experimental and modeling limitations, where only cement particles can be considered explicitly (readily knowing the initial water to cement ratio and size distributions), but one is (still) unable to explicitly account for nanometer-sized C-S-H nuclei that increase in amount with the cement hydration process and that may bridge the cement particles. This leads to the changed total displacement values of cement particles and subsequently increases the absolute strength of forces between cement particles, which complicates the description of actual real particle forces and their correlation to van der Waals forces or C-S-H bridges. Yuan et al. [17] assume that the displacement due to the measured small critical strain from SAOS measurement might be associated with the distance that is needed to overcome the vdW force attraction rather than only C-S-H bonding. Thus, SAOS as a non-destructive measurement technique in the linear viscoelastic domain (LVD) and is especially interesting as it provides information about the percolated network (rigid) structure, as well as forces responsible for the fragility of such a network.

### 2.2. Mathematical Models for Yield Stress Description

After quantifying $\tau_{y}$ experimentally, the employment of predictive microstructural-based models, such as Kapur’s model [15] from 1997 incorporating the calculation of apparent yield stress in a percolated network structure containing inter-particle bond orientation probability and YODEL [14], opens new avenues to investigate key fundamental parameters. Although they are well-used models to predict the yield stress of inert suspensions, adapted only partially to cement pastes [18,19,20], such mathematical tools have not yet been employed to predict yield stress obtained via SAOS measurements. Therefore, in this section, the background of the original Kapur’s and YODEL models are first summarized, followed by a newly proposed model based on an extension of Kapur’s model. We then compare the extended Kapur’s model and the original YODEL model to quantify the magnitude of the average attractive inter-particle forces responsible for $\tau_{y}$, and to check the hypothesis explaining their origin. Next, the theory behind all three models is presented in order to understand the underlying differences and their suitability for describing $\tau_{y}$ (obtained via SAOS). 

#### 2.2.1. Kapur’s Model

Kapur et al. [15] proposed a microstructural-based model based on Rumpf’s model [21] for the prediction of the tensile strength of mono-sized agglomerated particles by extending it to consider polydispersity. Both models calculate the stress as the product of the inter-particle force and the number of particle contacts per unit area. For flocculated size-distributed particles, Kapur’s model for yield stress is expressed as a product of the (total) number of particle contacts ($N_{k,l}$) between each pair of size classes *k*-*l* with the inter-particle attractive force ($F_{k,l}$):

(4) $$\tau_{y}=\frac{1}{6}\overset{n}{\underset{k=1}{\sum }}\overset{n}{\underset{l=k}{\sum }}N_{k,l}F_{k,l}$$

Thus, the product is summed over all *k*-*l* pairs, namely via a double loop over *k* and *l* particle size classes (*n* size classes in total). The attractive interactions between the two particles are initially attributed to van der Waals forces:

(5) $$F_{\mathrm{vdW}}=\frac{A_{0}\;a^{*}}{12\;H^{2}}$$

where the a* is the harmonic average of the radii, *A*_0_ is the Hamaker constant and *H* is the surface-to-surface separation distance. The number of contacts ($N_{k,l}$ in Equation (4)) is related to the coordination number *C*_k,l_, which is the mean number of particles of size *d*_k_ neighboring via pseudo-contacts with a central particle of size *d*_l_:

(6) $$N_{k,l}=n_{k}C_{k,l}\;w$$

where the factor w has value w = 1 if *k* ≠ l or w = ½ if *k* = l, while $n_{k}$ is the number of particles of size *d*_k_, calculated according to Kapur et al. [15], but here upgraded by following YODEL [14] to also consider $\phi_{perc}$:

(7) $$n_{k}=\frac{6\;\left(\phi -\phi_{perc}\right)\;}{\pi \;d_{k}^{2}}S_{k}$$

where $S_{k}$ is the fractional surface area of particles having size *d_k_*:

(8) $$S_{k}=\frac{\phi_{k}/d_{k}}{\sum_{l=1}^{n}\phi_{l}/d_{l}}$$

The percolation threshold represents a minimal solid volume required to exhibit a yield stress [14,18], after reaching a physical percolation steady state time. Below this threshold, the flocs do not form a (sufficiently) connected gel microstructure, and thus exhibit no yield stress. Thus, the introduction of the percolation threshold (in Equation (7)) reduces the number of (total) particles into an effective number of particles connected in the gel microstructure (*n*_k_ in Equation (7)), and is not considered to be a function of the initial cement suspension solid fraction (for the same cement) [18]. However, it depends on the plasticizer-induced dispersion degree [18] and possibly on the hydration degree, both of these effects not being considered in this paper.

Zhou et al. [22] showed that the initially introduced empirical dependence of the separation distance (*H* in Equation (5)) on solid volume fraction (in the original Kapur’s model [15]) is actually not needed. This is also demonstrated later by YODEL [14], emphasizing that all the microstructural contributions affecting the yield stress can be modeled as separate variables from forces.

#### 2.2.2. YODEL Model

Flatt and Bowen [14] proposed a yield stress model for suspensions, called YODEL, which is based on further microstructural (statistical geometrical) considerations of colloidal particle interactions. The YODEL model defines yield stress (τ_0_) as the beginning of flow when van der Waals bonds of percolated particles have orientation probability [14] to be fragmented. It describes it as a function of the magnitude of the maximal van der Waals force (Equation (5)) between particles, as follows:

(9) $$\tau ≅\frac{m}{d_{50\;}^{2}}F_{\mathrm{vdW}}\frac{\phi^{2}\left(\phi -\phi_{\mathrm{perc}}\right)}{\phi_{m}\left(\phi_{m}-\phi \right)}$$

In Equation (9), there are material-related and solid–water mixture variables. The three volume fractions are the solid (ϕ), percolation ($\phi_{\mathrm{perc}}$) and maximum packing ($\phi_{m}$) particle concentrations. The percolation volume fraction is a consequence of the interplay between Brownian motion (dispersive) and colloidal attractive forces between particles [5,11,18]. The cement material-related parameters are the non-retarded Hamaker constant (A_0_ = 1.6 10^−20^ J for C_3_S), while a* is the radius of curvature at the semi-contact points (~300 nm [18,19,20]), *d*_50_ is the cement particle average diameter and *H* is the surface-to-surface separation distance that depends on the dosage of admixtures. The factor *m* in Equation (9) depends on the particle size distribution and can be calculated according to the original YODEL model [14], as described in the next (theoretical) section. The model accurately predicts the dependence of yield stress on the solid volume fraction of inert suspensions; however, the scaling of yield stress is not directly predicted from particle size, but from the radius of curvature at the point of particle (semi-)contact. 

The starting point for the derivation of the YODEL model is based on the expression for the increase in the effective volume fraction of solids, or the equivalent decrease in maximum packing density, due to the flocculation of particles:

(10) $$\Delta \phi =\overset{n}{\underset{k=1}{\sum }}\overset{n}{\underset{l=k}{\sum }}N_{k,l}f_{k,l}\Delta v_{k,l}$$

which is calculated as a (double) product of the number of contacts (*N_k,l_*, between particle sizes *k* and *l*) with the subsistence probability (*f_k,l_*) and with the volume increase of each particle pair ($\Delta v_{k,l}$), summed over all particle size classes. The YODEL model is based on an assumption to yield when the true solid volume fraction is equal to the apparent maximum packing [14], i.e., $\Delta \phi =\phi -\phi_{m}$. By inserting this condition in the above Equation (10) and writing it explicitly for shear stress (hidden in the subsistence probability expression [14]), we obtain the YODEL model in Equation (9).

The factor *m* in Equation (9) depends on the particle size distribution and can be calculated according to the original YODEL model, as follows:

(11) $$m=\frac{3.6}{\pi^{4}}\overset{n}{\underset{k=1}{\sum }}\phi_{k}\overset{n}{\underset{l=1}{\sum }}C_{k,l}^{max}\frac{\Delta v_{k,l}}{b_{k}^{3}}g_{k,l}$$

The theoretical background behind this equation is detailed in [14], and here only the calculation of the parameters is briefly outlined for the sake of completeness and the reproducibility of the modeling results presented in this paper. The double sum composes a matrix of interactions between all particle size classes, namely between each size class *d_k_* (first loop with the index *k*), having a volume fraction $\phi_{k}$, with all other size classes d_l_, where the index l defines a second loop. The factor $C_{k,l}^{max}$ in Equation (11) is defined as a coordination number at the maximum packing density (using β = 0.4; [23]):

(12) $$C_{k,l}^{max}=\beta \;S_{l}\;\left(\frac{2\left(d_{l}+d_{k}\right)}{d_{l}+d_{k}-\sqrt{d_{k}\left(d_{k}+2d_{l}\right)}}\right)$$

A factor $g_{k,l}$ (in Equation (11)) is defined as:

(13) $$g_{k,l}=2\frac{\tilde{b}}{0.5\;d_{50}\left(b_{k}^{2}+b_{l}^{2}\right)}$$

where *b* is the particle radius normalized to the median volume diameter (*d*_50_), calculated as follows:

(14) $$b_{k}=\frac{a^{*}}{0.5\;d_{50}}$$

and $\tilde{b}$ in Equation (13) is a harmonic average of the interacting particle pair, which in case of cement suspensions was related to a characteristic radius of curvature (a*) defining the irregular surface of cement grains (to be fixed to several hundred nm, namely 300 nm [18,19,20]). The last parameter needed to calculate *m* from Equation (11) is the increase in the effective volume fraction due to flocculation, $\Delta v_{k,l}$. In the original YODEL model, three geometrical choices were proposed to calculate the effective volume fraction, which is calculated for each pair of interacting particles. In this paper, we take the first option from YODEL, a so-called truncated cone without particle fraction, for which the volume increment is calculated as follows:

(15) $$\Delta v_{k,l}=\frac{4\pi }{3}\frac{{\left(2b_{k}b_{l}\right)}^{2}}{\left(b_{l}+b_{k}\right)}$$

The original alternative version of the YODEL model, which takes the particles percolation into account in the coordination number determination, has a different form than the one used so far in modeling cement suspensions [18,19,20] (comparison of Equation (9) with Equation (16)),

(16) $$\tau ≅\frac{m}{d_{50\;}^{2}}\;F\frac{\phi \;{\left(\phi -\phi_{perc}\right)}^{2}}{\phi_{m}\left(\phi_{m}-\phi \right)}$$

The origin of this alternative expression is that the number of contact points in Equation (16) considers only the contacts of particles involved in the percolating network, while Equation (9) overestimates this number, as it neglects the percolation phenomena.

In the application of the YODEL model to cement suspensions, percolation threshold, together with the surface-to-surface separating distance, depend on the amount of used superplasticizers. Although simplified, the YODEL model was demonstrated to be very successful in predicting the key parametric dependencies of cement suspensions [17,18,19,20]. Considering estimations of the average separation distance *H* to be around 1.6 nm [18,19] (1.3 nm [17]) without, and 5 nm with, plasticizers [18,19] the value of the *m* pre-factor was used as a fitting parameter. Therefore, to date, the application of such a simplified YODEL model to cementitious materials [17,18,19,20] did not consider the particle interaction effects explicitly. However, we attempt to consider all the microstructural-based salient features of the original models, developed for simple (inert) particulate suspensions [14,15], and extend them for the SAOS yield stress of cement suspensions. Lecompte et al. [19] made the latest adaption of the simplified YODEL model for cement suspensions, by considering the hydration reaction effects to describe the structural buildup at rest.

The adaption of the YODEL model in this paper was implemented to quantify the magnitude of the average attractive force (*F*), but it does not (yet) explicitly model the origin of this force. The attractive force (*F*) is considered as an averaged value, corresponding to the cement particles’ average radius of curvature (a*), i.e., for every interaction between *k* and *l* cement particle size classes. This averaging means that, as a first approach, the force does not explicitly depend on the cement particle size (for cement particles having *d* ≥ a*) at the microscopic size class level, and that the effect of polydispersity is only implicitly included via the double sum over all relevant bonds, i.e., by considering the number of particle contacts. Thus, it should be kept in mind that the accuracy of the inversely estimated averaged interaction force parameter using the approach proposed in this paper mainly depends on the accuracy of the statistical microstructural description (i.e., via the prediction of the coordination numbers). The effects of nucleate particles, such as C-S-H and ettringite, are not explicitly included, but are considered implicitly in the estimated magnitude of the average force acting between cement particles.

#### 2.2.3. Yield Stress Model Based on Inter-Particle Bond Breaking Probability (BreakPro)

A new model for yield stress, proposed here, leans on Kapur’s yield stress model [15]. In short, we are extending Kapur’s model (Equation (4)) by considering the percolation threshold and (non-)subsistence probability, i.e., the effects of bond orientation, following the YODEL approach:

(17) $$\tau_{y}=\frac{1}{6}\overset{m}{\underset{k=1}{\sum }}\overset{m}{\underset{l=k}{\sum }}N_{k,l}F_{k,l}\left(1-f_{k,l}\right)$$

The major novelty of our approach, in Equation (17), is the introduction of the probability of broken bonds, namely the factor (1−$f_{k,l}$) which is calculated by extending the subsistence probability ($f_{k,l}$), as proposed by Flat and Bowen [14]. While YODEL is based on the calculation of the effective solid volume increase due to the number of unbroken bonds, Kapur’s model is based on the total number of broken bonds, here improved by reducing $N_{k,l}$ with a factor (1−$f_{k,l}$). The subsistence probability [14] defines the fraction of pair configurations that cannot be separated for a given shear stress. It accounts for a random orientation of individual pairs, where the stress is null if a pair is aligned in the direction of applied stress (e.g., in x-direction), while it is maximum if the pair is vertical to this direction (e.g., in y-direction). Thus, in the YODEL model, the subsistence probability is proportional to the maximum force and inversely proportional to the applied shear stress. The mathematical treatment of the subsistence probability is detailed later in Section 2.2.4, together with numerical implementation. The coordination number is considered to be a linear function of the solid volume fraction, following YODEL [14] to enable a better comparison of the models, focusing on the effects of the newly introduced BreakPro features (and disregarding any differences in calculation of the coordination numbers):

(18) $$C_{k,l}=\frac{C_{k,l}^{max}}{\phi_{max}}\;\phi$$

where the factor $C_{k,l}^{max}$ (in Equation (18)) is defined as a coordination number at the maximum packing density and calculated according to Equation (12).

The substitution of Equations (6) and (7) into Equation (17) finally result in the newly proposed mathematical model for yield stress:

(19) $$\tau_{y}=\left(\phi -\phi_{perc}\right)\frac{\phi \;F}{\pi \;\phi_{m}}\;\overset{m}{\underset{k=1}{\sum }}\frac{S_{k}}{d_{k}^{2}}\overset{m}{\underset{l=k}{\sum }}C_{k,l}^{max}\left(1-f_{k,l}\left(\tau_{y}\right)\right)$$

where $C_{k,l}^{max}$ is calculated according to Equation (12) and $S_{k}$ following Equation (8). As detailed above (in this section), the subsistence probability [14] defines the fraction of pair configurations that cannot be separated for a given shear stress, thus *f_k,l_*($\tau_{y}$). For this reason, our model results in an implicit Equation (19) that needs to be solved for $\tau_{y}$.

The newly proposed yield stress model (Equation (17) in a most general form, named BreakPro) extends Kapur’s model (Equation (4)) by introducing the inter-particle bond breaking probability (i.e., bond orientation effects), namely the factor (1 − $f_{k,l}$) and the percolation threshold ($\phi_{perc}$ parameter introduced in Equation (7)). The major difference is that the YODEL model calculates yield stress from unbroken bonds (subsistence probability *f*) and we introduced the broken ones (1 − *f*). Moreover, the subsistence probability is solved in a more accurate way by giving a new analytical solution, as detailed in Section 2.2.4.

#### 2.2.4. Numerical Implementation

The theoretical background for calculating subsistence probability is given in [14], where the final equation used in the YODEL model is actually based on a simplified expression, due to the complexity of the analytical solution. However, the simplification is no longer valid for our approach, as it cannot assure computational convergence when solving Equation (19). This is due to the different modeling approaches (our model vs. YODEL). Our model results in an implicit Equation (19), as the subsistence probability (*f*) is also a function of unknown $\tau_{y}$, which needs to be solved for. Therefore, a numerical solver (*fzero* function in Octave/Matlab) is employed in this paper to solve the resulting non-linear Equation (19) for $\tau_{y}$.

The average subsistence probability in its original derivation [14] is given by following integration over the initial orientation angles (ϑ) of particle pairs:

(20) $$f_{k,l}=1-\frac{4}{\pi^{2}}\int_{0}^{\pi /2}\arctan\left(\frac{\alpha_{k,l}}{\cos\left(\vartheta \right)}\right)d\vartheta$$

where $\alpha_{k,l}$ is not dependent on the angle ϑ (important to solve the integral) and is defined as:

(21) $$\alpha_{k,l}=\frac{\pi \;\tau \;4\left(d_{k}^{2}+d_{l}^{2}\right)}{3\;F\left(\phi -\phi_{perc}\right)}$$

In this paper, we found an analytical solution for the integral in Equation (20), by means of *Wolfram Mathematica*, which enabled us to numerically calculate Equation (20) as:

(22) $$f_{k,l}=\frac{4\;HypGeomFun\left(\left[\frac{1}{2},1,\;1\right],\;\left[\frac{3}{2},\frac{3}{2}\right],\frac{-1}{\alpha_{k,l}^{2}}\right)\;}{\pi^{2}\;\alpha_{k,l}^{2}}$$

where the hypergeometric function (*HypGeomFun*) is defined as:

(23) $$HypGeomFun\left(\left[\frac{1}{2},1,\;1\right],\;\left[\frac{3}{2},\frac{3}{2}\right],\frac{-1}{\alpha_{k,l}^{2}}\right)=\overset{\infty }{\underset{n=0}{\sum }}\left(\frac{{\left(\frac{1}{2}\right)}_{n}{\left(1\right)}_{n}{\left(1\right)}_{n}}{{\left(\frac{3}{2}\right)}_{n}{\left(\frac{3}{2}\right)}_{n}}\right)\left(\frac{-\alpha_{k,l}^{-2n}}{n!}\right)$$

where (*x*)*_n_* factors represent Pochhammer symbols, which are defined via gamma (Г) functions as:

(24) $${\left(x\right)}_{n}=\frac{Г\left(x+n\right)}{Г\left(x\right)}$$

The inverse parameter estimation of the average inter-particle force (*F*) in model Equation (19) is obtained by using the *lsqnonlin* optimization function in GNU Octave (version 5.2.0, free software) and Matlab (version R2019a, The Mathworks Inc., Natick, Massachusetts, USA). 

The presented way of mathematical derivation enabled us to develop a new approach to quantify the yield stress $\tau_{0}$, which subsequently could be compared to the experimentally investigated yield stress $\tau_{y}$ at the smallest critical strain obtained by SAOS measurements, and subsequently correlate and calculate it as a function of the water-to-cement ratio.

## 3. Materials and Methods 

Oscillatory rheometric measurements using a high-sensitivity rheometer and an SAOS protocol enabled us to measure the yield stress at the first critical strain beyond the linear viscoelastic region. The critical strain was defined as the limit of the linear viscoelastic region where the storage modulus is still constant and independent of the applied strain amplitude [17].

### Mix Design and Sample Preparation

An ordinary Portland cement (OPC), CEM I 42,5 R, exhibited the following phase composition: 55.8 mass% C_3_S, 14.6% C_2_S, 10.9% C_3_A, 7.4% C_4_AF and 5.3% sulfate carriers; specific surface: Blain 3615 cm^2^/g, and BET 12,350 cm^2^/g; and particle size distribution: *d*_50_ = 14.8 μm, *d*_10_ = 1.5 μm and *d*_90_ = 44.6 μm, as detailed further in [24]. Demineralized water with a temperature of 5 °C was used for an adjusted paste temperature of 20 °C. The temperature of the cement paste was controlled at 20 ± 1 °C during testing. Higher solid volume fractions in cement pastes lead to higher yield stresses due to lower (initial) particle distances and thus stronger agglomeration processes. Thus, for the investigation of the effect of solid volume fraction on the apparent yield stress $\tau_{\gamma }$, three different solid volume fractions, i.e., $\Phi_{act}$ = 0.41, 0.44 and 0.48, were tested. The pastes are named according to their solid volume fraction. The mixture proportions for all test series are given in Table 1.

The cement pastes were prepared using a drilling machine with a four-bladed propelling screw. The mix program was set to an initial mixing of 30 s after water addition followed by one minute of mixing at high shear intensity, namely 1700 rpm. The paste then was left to rest for 30 s and again mixed for one minute at high shear intensity (3 min in total). 

For the rheological measurements, an Anton Paar MCR 502 rheometer (Anton Paar GmbH, Graz Austria) with serrated plates (diameter = 50 mm) was used. The gap between the plates was set to 1 mm for homogenized sample conditions over the whole gap; the serrated plates, meanwhile, prevented wall slip. The Anton Paar MCR rheometer is a stress-controlled rheometer. A twin-drive option still allows the setting of a controlled strain. The amplitude sweep was started at an initial shear strain of $\gamma =1\times 10^{-5}$. The strain sweep oscillated for five data-decades with ten data points per data-decade, resulting in 51 data points in total. The critical strain for cement pastes is generally assumed to be between $\gamma =1\times 10^{-5}$ and $\gamma =1\times 10^{-4}$. The critical strain values are determined according to the standards ISO 6721-10 and EN/DIN EN 14770, namely by selecting the tolerance range of 5% deviation for G′ around the plateau value (calculated as an average value of the data points before reaching the targeted deviation). The frequency during the amplitude sweep was set to ω = 10 rad/sec, which was found to be an appropriate frequency for conducting oscillatory measurements on cement pastes.

## 4. Results and Discussion

### 4.1. SAOS Measurements

The results of the strain sweep measurements for all test series are given in Table 2. The storage modulus $G^{\prime }$ in (Pa), loss modulus $G^{\prime \prime }$ in (Pa), the critical strain at the end of the linear viscoelastic regime $\gamma_{l}$ in (%) and the calculated yield stress $\tau_{\gamma }$ in (Pa) are presented in Table 2.

**Figure 1:**
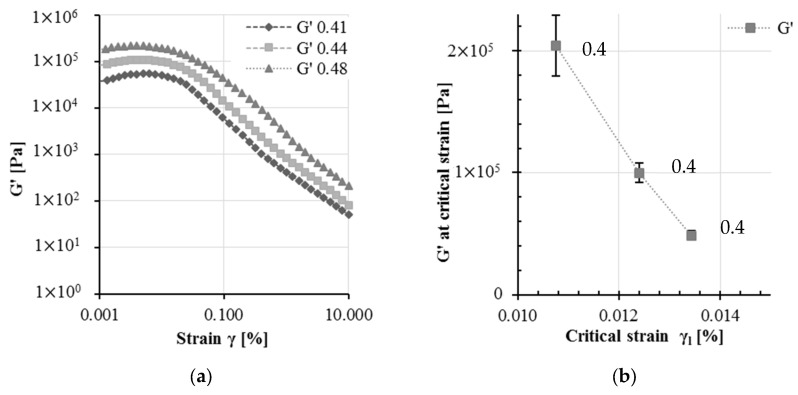
Results of strain sweep measurements for solid volume fractions of 0.41, 0.44 and 0.48: (**a**) the storage modulus $G^{\prime }$ versus strain and (**b**) the storage modulus for each test series dependent on the critical strain (error bars indicates the magnitude of a standard deviation).

Figure 1 presents the results of the oscillatory measurements. The average SAOS measurements of three repetitions are shown for each test series. The standard deviation was calculated for a small sample number using the Gaussian distribution. Due to very low standard deviations, the measurements show a good reproducibility. Figure 1a shows the storage modulus $G^{\prime }$ over strain values. In the linear viscoelastic region, up to about 0.01%, the storage modulus $G^{\prime }$ is observed to increase remarkably when increasing the amplitude. This could be explained by the cement paste reactivity and physical agglomeration processes. The low strain values are not sufficient to break any flocs. A slight increase in storage modulus within the LVE region can be observed for all test series. It can be assumed that, even during the short time range, a slight thixotropic structural buildup of particles caused by physical attraction forces takes place.

When surpassing a critical strain $\gamma_{l}$, the storage modulus decreases with increasing strain, which corresponds to the end of the linear viscoelastic domain. Above this critical strain, the breakage of the bonds within the network structure of the agglomerated cement particles takes place. Thus, the material response to an applied strain is not linear and no longer corresponds to Hookean material behavior, but reacts non-linearly to increasing strain. Figure 1b shows the storage modulus for each test series dependent on the critical strain $\gamma_{l}$: The test series 0.41 possesses the weakest stored energy within the particle network with an average storage modulus of 48.699 Pa. The storage modulus of the paste 0.44 possesses twice the strength, with 99.874 Pa. The highest value is apparent for the paste 0.48 with 204.267 Pa. These results correspond to results on SAOS measurements in previous research [11,16,25] and also correspond to theoretical basics that state higher network strength with increasing solid volume fraction. Meanwhile, the critical strain $\gamma_{l}$ desreases with increasing solid volume fraction ϕ. For the test series 0.48, the critical strain is 0.0108%. The value increases to 0.0124% for 0.44 and 0.0134% for 0.41. The observed trend of the critical strain upon the solid volume fraction depends on the destruction of the floc microstructure [26]. A lower solid volume fraction of particles thus contains a higher critical strain, because the gel can be assumed to be less fragile.

**Figure 2:**
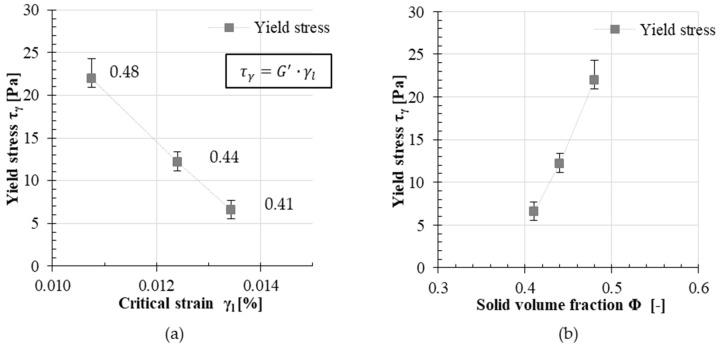
Calculated yield stress values of the test series 0.41, 0.44 and 0.48: (**a**) dependent on measured critical strain $\gamma_{l}$ and (**b**) dependent on solid volume fraction Φ (error bars indicate the magnitude of a standard deviation).

Still, all values for the critical strain are measured in similar regions. The main difference can be observed in calculating the yield stress $\tau_{\gamma }$ for each test series. Figure 2a presents the calculated yield stress for each test series dependent on its smallest critical strain value$\;\gamma_{l}$. The yield stress $\tau_{\gamma }$ was calculated according to Equation (3). In Figure 2b, the measured yield stresses $\tau_{\gamma }$ at the smallest critical strain $\gamma_{l}$ are plotted as a function of the solid volume fraction.

### 4.2. Modeling Approach 

**Figure 3:**
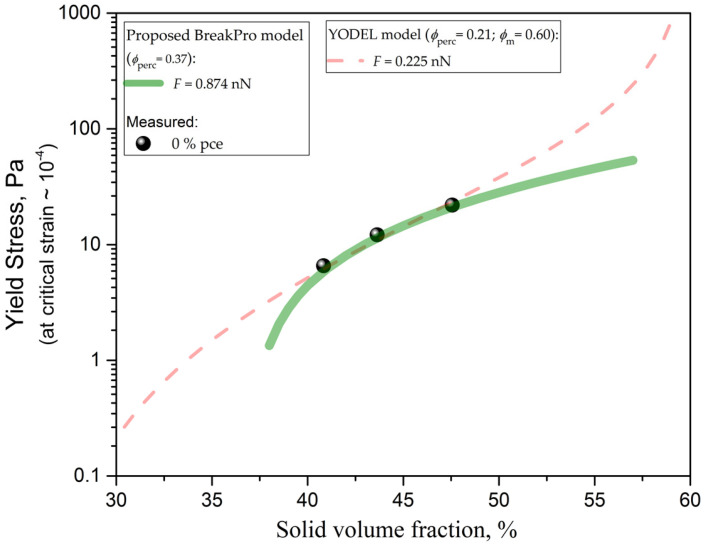
Correlation of computed yield stress by the proposed modeling approach (YODEL and BreakPro models) and experimental results in SAOS measurements for 0.41, 0.44 and 0.48 solid fractions.

The microstructural-based yield stress models were employed to quantify the magnitude of the average attractive force (*F*) between cement particles. This force is considered as an averaged value, in terms of the cement particles’ average radius of curvature (a*). Thus, one should always be aware that the accuracy of the inversely estimated averaged interaction force parameter using the approach proposed in this paper mainly depends on the accuracy of the employed statistical microstructural description. The inverse modeling, i.e., parameter estimation approach, resulted in the following values for the magnitude of the average attractive force (Figure 3): *F* = 0.874 nN (BreakPro model) versus *F* = 0.225 nN for YODEL. By testing the model predictions as a function of a solid volume fraction, we demonstrate the capabilities of the two modeling approaches, i.e., comparing the validity of YODEL assumptions with our newly proposed BreakPro model. 

When comparing the modeling results (Figure 3), the forces estimated using the BreakPro model were found to be 3.9 times higher than with YODEL. Moreover, the percolation threshold obtained by YODEL turned out to be significantly lower (21%) than the one found by BreakPro (37%). This could be attributed to the differing mechanisms between the rotational and oscillatory rheometry, explained namely by short-ranged (ion correlation, C-S-H bridging) forces versus long-ranged van der Waals displacements (as discussed later). The comparison of the modeling with the experimental results (Figure 3) shows a very good overall agreement between the measured and modeled values. This increases confidence in the BreakPro model, since the average force was used as the only fitting parameter. The percolation threshold parameter was fixed to a previously reported literature value also used in the conventional YODEL model (37% [18]) to fit the yield stress obtained by rotational rheometry (i.e., for flow onset at higher strains). The better agreement with the expected value for solid percolation [18] shows that the BreakPro modeling assumptions are better suited for the description of yield stress at SAOS critical strain than the YODEL model.

In order to obtain a good fit of the YODEL model with the experimental results, the percolation threshold had to be decreased by a fitting algorithm to as low as 21%. The physical meaning for such a lower percolation threshold is lacking, as theoretically similar percolation thresholds should be found when predicting rotational yield stress as for yield stress at SAOS critical strain. Here, it should be stressed that the percolation threshold depends on the floc microstructure, which also depends upon dispersion degree and aging. However, we are referring to the specific kinetic time where the initial (chemical) hydration is minimal (negligible) and still long enough to allow for the reaching of the physical percolation (steady state) time (as shown in [11]). In this state, the fact that the BreakPro percolation threshold is in good agreement with the one independently obtained via rotational rheometry and numerical simulations (using the random loose packing model) [18], indicates the preference for the newly proposed model. Therefore, the YODEL assumptions may not be representative for the yield stress at SAOS critical strain. In particular, in yield stress at SAOS critical strain, which represents the end of the LVE, a monotonous increase with solid volume fraction is expected until it reaches a finite maximum value for the maximum packing density. This is in contrast to the YODEL model assumption where the solid-to-liquid transition yield stress (at few % strains) predicts an infinite value at maximum packing density. The underlying physical reason for this is that the YODEL model describes the yield stress obtained via rotational rheometry based on the definition of imposing the no flow divergence at maximum packing density. In other words, this means that for a suspension at maximal packing density, it is not theoretically possible to initiate a flow at few % strains. Note that the model trend in Figure 3 has a logarithmic scale for yield stress, meaning that the slope is actually representing a power law increase. However, it is more important that this increase is much less pronounced in the BreakPro model than in the Yodel model, because the BreakPro model (at 10^−4^ strains) predicts a finite magnitude at maximal packing density (around 60% [18]). We argue that this represents the physical reality in a better way, compared to the Yodel model, which predicts an infinitive yield stress at maximal packing density. Yield stress at SAOS critical strain is defined as a critical shear stress limit below which the material behaves elastically with an undamaged microstructure. Thus, with an increase in strain above this critical one, the microstructure starts to be irreversibly damaged, resulting in a significant drop in elastic modulus (G′), and also in the maximal packing of particles (in analogy to the modeling of tensile strength [15,21]). Therefore, the negligible sensitivity of the BreakPro model to the maximum packing density depends only on the calculation of the coordination number (via Equation (18)) and stays negligible by eliminating the assumption to force the divergence of the yield stress at the maximal packing density, as imposed in YODEL (Figure 3). This difference is attributed to the differing mechanisms between the rotational and oscillatory rheometry, namely explained further by shorter range (ion correlation, C-S-H bridging forces) versus longer range (e.g., van der Waals) displacements.

The failure of the YODEL model to predict our measurements with reasonable percolation threshold values could arise from the short-ranged forces (C-S-H bridges or ion correlation-related attractive forces [11,12]) instead of the longer range colloidal (van der Waals) interactions. As concluded by Nachbaur et al. [25], the very small value of the critical strain (~0.01%) suggests that short-range forces bond the cement particles. Models proposed to predict the cohesion between C-S-H nuclei give significant attractive forces for separation distances below the 1 nm range [11,12]. On the other hand, van der Waals forces are significant in the separation distance range of up to several nm (at 5 nm, the force has 10% of the value at 2 nm), but its maximal attractive force is much less than that of C-S-H cohesion. However, due to the lack of a quantitative description of this cohesion force, they were not explicitly modeled in this paper, and this effort is recommended as a promising direction for future research.

It should be emphasized that the percolation threshold (and maximal packing density) parameter correlates with the estimated inter-particle force. However, this inter-dependency is strongest at the solid fraction threshold and rapidly loses its inter-dependency magnitude (i.e., decouples) with an increase (or decrease) in solid content. This results in the different shapes of the (yield stress versus solid fraction) curves for different combinations of the parameters, which enables the decoupling of the dependencies between parameters, even making it possible to attempt fitting both parameters simultaneously. To clarify the effect of the interdependencies between those parametric model choices, a parametric study was performed on our case study (Figure 3). For that purpose, we selected to impose an input variability of ± 1% (relative%; i.e., $\pm 0.01\;\phi_{m}$ or $\pm 0.01\;\phi_{\mathrm{perc}}$), as higher values would result in notable differences in the (miss-)fitting of the shape of the curve. The influence of the ± 1% variability in the choice of the maximum packing density resulted in ± 0.0% and ± 70% variability in the calibrated interaction forces using the BreakPro and YODEL models. The high dependency of the YODEL model is due to the model assumption that forces the divergence of the yield stress at the maximal packing density (Equation (16) and Figure 3). The influence of the ± 1% variability in the choice of the percolation threshold resulted in ± 7.5% and ± 0.0% variability in the calibrated interaction forces using the BreakPro and YODEL models. In the case of the YODEL model, the percolation threshold had to be over-fitted to an unrealistically low value (21% versus the expected 37%) in order to obtain good agreement with the experimental results (Figure 3). Such a low percolation threshold value thus resulted in a good decoupling from the estimated force parameter. However, a comparison of our results (Figure 3), obtained via SAOS rheometry, with the experimental results from the literature, obtained using rotational rheometry [18,20], indicates that the YODEL model well describes (with a realistic percolation threshold value of 37%) only much steeper (yield stress versus solid fraction) dependence, and for our results, one has to flatten the shape of the curve (Figure 3). The reasons for this are already discussed above, concluding that the BreakPro modeling assumptions are better suited for description of yield stress at SAOS critical strain than the YODEL model (which is suitable for data obtained by rotational rheometry).

The forces responsible for keeping the suspension rigid in the SAOS domain were found to be 5.57 (BreakPro model) and 1.43 (YODEL) times higher than the typical van der Waals forces. This comparison, i.e., the factor of difference, is related to the van der Waals force, calculated as 0.157 nN according to Equation (5), by inserting the average radius of curvature to be 300 nm and the separation distance to 1.6 nm, as a typical value for cements without the use of plasticizers [18,19]. The attractive forces responsible for the critical strain of 10^−4^ are commonly attributed to the C-S-H nuclei [9,25]. However, as these initial C-S-H nuclei are at the nanometer scale (1–2 nm) and initially have negligible volume, such particles could not be considered explicitly in the YODEL or BreakPro models. The initial amount of C-S-H (here within the first 4 min, but even longer in [27]) is questionable and below the detection limit of measurement techniques, and is thus not yet observed in the literature. The contribution of the cement cohesion, which results in a critical strain of about 10^−4^, is hypothetically ascribed in the literature to be due to the bridging of the cement particles by the initial C-S-H hydration product [9,25].

Next, we check the hypothesis whether ion correlation forces are responsible for the attraction between cement particles. The same origin could be assumed for the interactions between cement particles, i.e., without C-S-H bridging, due to the high pH and Ca^2+^ concentration, by assuming the same surface charges. The interaction between the highly negatively charged surfaces of cement and/or C-S-H particles in the presence of calcium divalent counter ions results in a strongly attractive forces due to ion correlations. Ion correlation forces were already suggested to be important for the stability of clays [28], and several important works demonstrate that they result in highly attractive forces that explain the cohesion of cement paste [11,12]. Traditional Derjaguin–Landau–Verwey–Overbeek (DVLO) theory fails to describe this electrostatic attraction, even qualitatively, as the electrostatic part of the DLVO can predict only repulsion. This is because the DLVO is based on the mean field approximation of the Poisson–Boltzmann model, which is limited to low surface potentials (i.e., low surface charges) and monovalent counter-ions. However, due to the high pH and reactivity of cementitious suspensions, the resulting high surface charges and the divalence of the calcium counter-ions, result in an attractive electrostatic contribution to the osmotic pressure. Therefore, other modeling approaches (e.g., Monte Carlo simulation methods [29]) are needed to model these attractive forces. The ion correlation forces are demonstrated experimentally by atomic force microscopy (AFM) measurements for C-S-H cohesion [30], resulting in a force of approx. 1 nN, between the two C-S-H surfaces with an interactive area of 64 nm^2^. In our modeling approach, the interaction surface can be considered as an averaged value, following a YODEL approach where the interaction force is calculated from the cement particles’ average radius of curvature (Equation (5)). Comparing AFM with our (BreakPro) force estimates, (64 nm^2^)·(0.874 nN)/(1 nN), resulted in the intersection surface area of 55.9 nm^2^. This suggests that either a very small amount of C-S-H particles is needed to induce attractive forces between cement particles, or alternatively, ion correlation origin does not require C-S-H, but could also form between the (hydrated) surfaces of cement particles with an interaction area of 55.9 nm^2^. This interaction area is in good agreement with the expected one based on the interaction of two radii of curvature of 300 nm (as proposed in [18,19] when employing a simplified YODEL model to cementitious materials).

Next, we demonstrate the effect of subsistence probability introduced in the BreakPro model to extend Kapur’s model in order to calculate the actual number of broken bonds by introducing a factor (1 − $f_{k,l}$). This model was already compared with YODEL, which is based on the calculation of the effective solid volume increase due to the number of unbroken bonds, thus having a factor $f_{k,l}$. The difference between these factors has a simple physical meaning, which is that the probability of unbroken bonds is directly related to the probability of the broken ones via *f*_broken_ = 1 − *f*_unbroken_. The subsistence probability defines the fraction of pair configurations that cannot be separated for a given shear stress, due to their unfavorable (statistical) orientation. We demonstrate the effect of subsistence probability, by using Kapur’s model, i.e., Equation (19), without a non-subsistence probability factor (1 − *f*), which resulted in a lower value of estimated forces, namely *F* = 0.460 nN. This result was not presented in Figure 3, as the fitted curve showed no significant difference with the plotted one using the BreakPro model. The only difference is in the magnitude of the estimated force, being a factor of 1.90 higher in BreakPro compared to Kapur’s model. This indicates that in the BreakPro model, approximately half of the particle bonds have a statistical alignment that allows for being broken, while the other half stays unbroken. In other words, the more correctly estimated value for the average force was doubled, as only half of the bonds were found to be broken due to their statistical orientations.

In ongoing and prospective research, we would like to outline a valuable comprehensive review, given by Bonn et al. [31], addressing the physical behavior of a broad range of yield stress materials in soft condensed matter. It emphasizes the role of relaxation time scales in the interplay between shear flow and aging behavior and encourages the usage of a microscopic theoretical description of the flow dynamics of yield stress materials [31,32]. However, the question of the yielding dynamics, even for simple non-reactive systems, still remains as an ongoing research topic, which should be explored, preferably by a combination of experimental and theoretical approaches, at microscopic and mesoscopic levels.

## 5. Conclusions

In this paper, average attractive forces between cement particles were estimated using yield stress microstructural-based models and SAOS oscillatory rheological measurements. The newly proposed yield stress model, named BreakPro, extended Kapur’s model by introducing the inter-particle bond breaking probability (i.e., bond orientation effects) and the percolation threshold. The major difference is that YODEL calculates yield stress from unbroken bonds (subsistence probability *f*), and we introduced the broken ones (1 − *f*). Moreover, the subsistence probability is solved in a more accurate way by giving a new analytical solution. To demonstrate the capabilities of the proposed modeling approach, the results were compared with measured yield stresses obtained by SAOS.

Based on the results presented in this paper, the following conclusions can be drawn:

- The calibration of the BreakPro model to the experimental values resulted in an estimated percolation threshold of 37%, in agreement with previous results from the literature [11], while YODEL resulted in a significantly lower calibrated value of 21%. This was attributed to the differing mechanisms between the rotational and oscillatory rheometry, explained namely by shorter range (ion correlation, C-S-H bridging forces) versus longer range (e.g., van der Waals) displacements. This demonstrates that the BreakPro modeling assumptions are better suited for the description of yield stress at SAOS critical strain than the YODEL model.
- The forces estimated using inverse BreakPro and YODEL modeling approaches were found to be 5.57 and 1.43 times higher, respectively, than typical van der Waals forces (Equation (4)).
- Comparing the BreakPro force estimates with the literature values for C-S-H cohesion forces, obtained by AFM, resulted in an average inter-particle intersection surface area of 55.9 nm^2^. This implies that either a very small amount of C-S-H particles is needed to induce such strong attractive forces between cement particles (after 4 min of hydration), or that ion correlation forces do not require C-S-H, but could also form directly between the (hydrated) surfaces of cement particles.
- As the obtained experimental results are specific to the applied mixing energy and hydration time, in future research, the effect of mixing energy and hydration time on the estimated model parameters should be investigated.

## Figures and Tables

**Table 1:** Cement paste mixtures.

Mixture	w/c Ratio	Solid Volume Fraction Φ	Cement (kg/m^3^)	Water (kg/m^3^)
0.41	0.46	0.41	1275.1	590.0
0.44	0.41	0.44	1368.4	560.0
0.48	0.35	0.48	1492.8	520.0

**Table 2:** Results from small amplitude oscillatory shear (SAOS) measurements.

Mixture	G′ (Pa)	G″(Pa)	$\gamma_{l}\;(\%)$	$\tau_{y}\;(\mathrm{Pa})$
0.41	48,699	16,886	0.0134	6.52
0.44	99,874	13,319	0.0124	12.38
0.48	204,267	15,336	0.0108	22.06
